# Superficial Neuromodulation in Dysautonomia in Women with Post-COVID-19 Condition: A Pilot Study

**DOI:** 10.3390/brainsci15050510

**Published:** 2025-05-16

**Authors:** Alberto Melián-Ortíz, Eduardo Zurdo-Sayalero, Sara Perpiñá-Martínez, Antonio Delgado-Lacosta, Carmen Jiménez-Antona, Josué Fernández-Carnero, Sofía Laguarta-Val

**Affiliations:** 1 Faculty of Nuring and Physiotherapy Salus Infirmorum, Universidad Pontificia de Salamanca, 28015 Madrid, Spain; amelianor@upsa.es (A.M.-O.); ezurdosa@upsa.es (E.Z.-S.); sperpinama@upsa.es (S.P.-M.); ardelgadola@upsa.es (A.D.-L.); 2 Department of Physical Therapy, Occupational Therapy, Rehabilitation and Physical Medicine, Faculty of Health Sciences, Universidad Rey Juan Carlos (URJC), 28922 Alcorcón, Spain; carmen.jimenez@urjc.es (C.J.-A.); sofia.laguarta@urjc.es (S.L.-V.); 3 Cognitive Neuroscience, Pain and Rehabilitation Research Group (NECODOR), Faculty of Health Sciences, Universidad Rey Juan Carlos (URJC), 28933 Madrid, Spain

**Keywords:** post-COVID-19 condition, dysautonomia, nervous system, heart rate variability, superficial neuromodulation

## Abstract

Post-COVID-19 condition involves persistent symptoms after acute infection, often linked to dysautonomia, which affects heart rate variability, pain perception, fatigue, and sleep. Superficial neuromodulation has been proposed as a treatment. **Objective:** To assess the effects of superficial neuromodulation on symptoms, sleep quality, and autonomic function in post-COVID-19 condition patients. **Methods:** A pilot study was conducted based on a triple-blind randomized controlled trial methodology involving 16 female participants. The experimental group received neuromodulation, while the control group used a placebo device. The intervention spanned 15 sessions over two months. Primary outcomes included heart rate variability, pain threshold, cortisol levels, fatigue, sleep quality, and quality of life, analyzed using repeated-measures ANOVA. **Results:** Both groups improved over time. Heart rate variability (SDNN) increased in the experimental group (30.42 to 39.11 ms) but decreased in controls (31.88 to 28.73 ms) (*p* < 0.05). Pain threshold at C5–C6 improved in the experimental group (2.1 to 3.5 kg/cm^2^) but remained stable in controls (*p* = 0.032). Fatigue decreased significantly in both groups (*p* = 0.002). Sleep quality improved, with Pittsburgh Sleep Quality Index scores decreasing similarly in both groups. Cortisol levels increased, with a non-significant trend favoring controls. **Conclusions**: While improvements were seen, both groups benefited, suggesting a possible placebo effect. Superficial neuromodulation appears safe, but further studies with larger samples are needed to confirm efficacy.

## 1. Introduction

A significant percentage of individuals (20–90%) who recovered from the acute phase of COVID-19 began reporting a range of clinical manifestations—both subjective and objective—that persisted beyond three weeks and even three months post infection. This condition, termed post-COVID syndrome, long COVID (LC), or persistent COVID, was identified as a new health challenge [1].

Currently, the WHO recommends referring to the aforementioned condition as a “post-COVID-19 condition”, since this name does not attribute causality or duration, and there are already specific ICD-10 (U09) and ICD-11 (RA02) codes to identify them [2].

In 2020, following WHO guidelines, the Spanish Society of General Practitioners and Family Physicians (SEMGF), along with 48 other medical societies, published a clinical guideline for persistent COVID [3,4]. This guideline defined it as a “multi-organ symptomatic complex affecting patients who have had COVID-19 and continue to exhibit symptoms beyond the acute phase of the disease, persisting for 4 or even 12 weeks” [3].

Up to 82% of COVID-19 patients report persistent symptoms, with the most common being: fatigue (55%), dyspnoea (42%), headache (37%), memory loss (34%), sleep disturbances (31%), anosmia or ageusia (26%), and concentration difficulties (28%) [5,6].

Regarding gender, the female sex appears to be a significant risk factor for developing this condition, as demonstrated by a study conducted by the Health Science Center at the University of Texas in San Antonio (United States). This large-scale investigation (*n* > 12,000) revealed that women have a 31% higher probability of developing the condition compared to men. Furthermore, female patients exhibited both a broader spectrum of symptoms and greater symptom severity. This risk elevation persisted even after controlling for potential confounders including race, ethnicity, viral variant, infection severity, and non-medical social determinants of health. The study highlighted that women aged 40–55 years demonstrated the highest propensity for developing the condition [7].

Regarding possible causative mechanisms, we found [8]: Excess inflammation: It is the main candidate, since high and abnormal levels of inflammation are observed in many patients; Autoimmune response: A large number of autoantibodies have been found in patients with post-COVID-19 conditions. And certain autoimmune diseases, such as lupus and rheumatoid arthritis, commonly cause fatigue and digestive problems; Nervous system problems: Dysautonomia (DNS), which affects blood flow, including to the brain, so it can cause fatigue, headaches, brain fog, and exercise intolerance and Blood clots and vascular damage: Many patients have high levels of D-dimer in the blood, and with prophylactic anti-coagulation they improve. Widespread clots were found in autopsies of people who died of COVID-19. Sleep quality disturbances appear to have multifactorial causes, including post-traumatic sequelae from the pandemic, oxygen saturation issues due to pulmonary complications, and the demonstrated neurotropism of coronaviruses [9].

Many of these aforementioned manifestations are associated with DNS, a neurological disorder caused by dysregulation of the autonomic nervous system (ANS) [5]. In many hospitalized patients with mild to moderate symptoms, parasympathetic dominance (PNS) has been observed compared to healthy controls [10], with some studies referring to this phenomenon as “exhaustion” of sympathetic tone due to ANS dysregulation. One hallmark of this DNS is the alteration of heart rate variability (HRV), which could explain the persistence of symptoms in these patients [11]. HRV is a non-invasive biomarker providing insights into ANS function and cardiovascular health. Its analysis reflects beat-to-beat variability of heart rhythms and offers information on the autonomic modulation of heart rate [12]. Studies have reported reduced HRV in post-COVID-19 condition patients, with this reduction correlating to greater symptom severity. Thus, strategies aimed at improving ANS function and HRV may help mitigate persistent health effects and improve patient quality of life [12,13].

The primary objective of this study was to mitigate symptoms, improve sleep quality and quality of life, and reverse DNS in post-COVID-19 condition patients. A secondary objective was to analyze and evaluate changes in mechanical sensitization by measuring Pressure Pain Threshold (PPT), cortisol levels, and HRV fluctuations.

To achieve the study’s objectives, non-invasive neuromodulation was selected as the physiotherapy technique targeting the ANS. This gentle, non-aggressive method employs biphasic microcurrents with oscillating frequencies between 1.14 and 14.29 Hz (depending on the program), intensities ranging from 0.1 to 0.9 mA, and low voltage (±3 V). These currents are applied to distal nerve endings of the extremities using 24 electrodes (six per extremity) distributed across both wrists and ankles, creating a circulating bioelectric circuit to stimulate the ANS.

## 2. Materials and Methods

A triple-blind randomized controlled trial (NCT05681455) was conducted following approval from the Ethics Committee of the University Hospital Fundación Alarcón (Madrid, Spain) under protocol number 22/32. The study was carried out at the Clinical University of the Faculty of Nursing and Physiotherapy Salus Infirmorum, Pontifical University of Salamanca, Madrid Campus.

To calculate the sample size, the estimated effect size for the primary outcome measures was 0.25. Using a statistical power of 0.95 and an alpha error of 0.05, a minimum sample size of 60 participants was calculated using G*Power software (version 3.1.7). Accounting for a 10% dropout rate, the final target sample size was 60 patients (*n* = 60), with 33 participants allocated to each group to prevent loss.

Participants were recruited via an email invitation sent to the AMACOP Association (Persistent COVID Patients Association). Nineteen individuals responded and attended a consultation with an internist, who assessed their eligibility according to the study criteria. Since the target sample size was not achieved, the study was conducted as a pilot, linked to previously published trials by the same research team [14,15,16].

We included women diagnosed with post-COVID-19 condition for more than one year; central sensitization symptoms. We excluded women above 60 years or below 18 years; previous surgical treatment; history of spinal trauma or cervical whiplash; current pregnancy; diagnosed fibromyalgia; implanted pacemaker or drug-dispensing electronic pump; skin sensitivity disorders.

One participant was excluded due to meeting an exclusion criterion. The remaining 18 participants were randomized into two groups: nine in the experimental group (EG) and nine in the control group (CG). Randomization was conducted using online software (GraphPad Software, Inc., La Jolla, CA, USA), version is 10.4.2. All participants provided informed consent. Before intervention initiation, two CG participants withdrew due to recommendations from their primary care physicians, leaving seven participants in the CG.

To carry out the intervention, 6 NESA XSIGNAL^®^ superficial neuromodulation devices were available (three for each group); only their manufacturer (NESA WORLD, San Sebastián, Spain) was aware of the three that worked and the three that were placebo.

Intervention Protocol:

The study lasted two months, with 15 sessions of neuromodulation scheduled over alternating days (three times a week) in the same time slots. EG participants were treated in a separate room from CG participants.

The EG received the experimental NESA XSIGNAL^®^ device, while the CG received a placebo device. The placebo devices functioned identically in appearance, with operational lights mimicking the experimental devices. As the applied microcurrents were imperceptible, participants were unaware of their group allocation.

The treatment targeted the ANS centrally, with directional electrodes focused on the C7 cervical vertebra and intensity set to “Low 3 V”.

The protocol followed 3 phases: The first phase consisted of two sessions conducted with programs P1 (30 min), P7 (15 min), and P8 (15 min) to avoid adverse effects. The second phase consisted of six sessions with programs P5 (30 min), P7 (15 min), and P8 (15 min) focused on addressing fatigue and ANS. And the last phase was seven sessions with programs P6 (15 min) and P7 (45 min) aimed to reduce pain perception.

**Figure 1:**
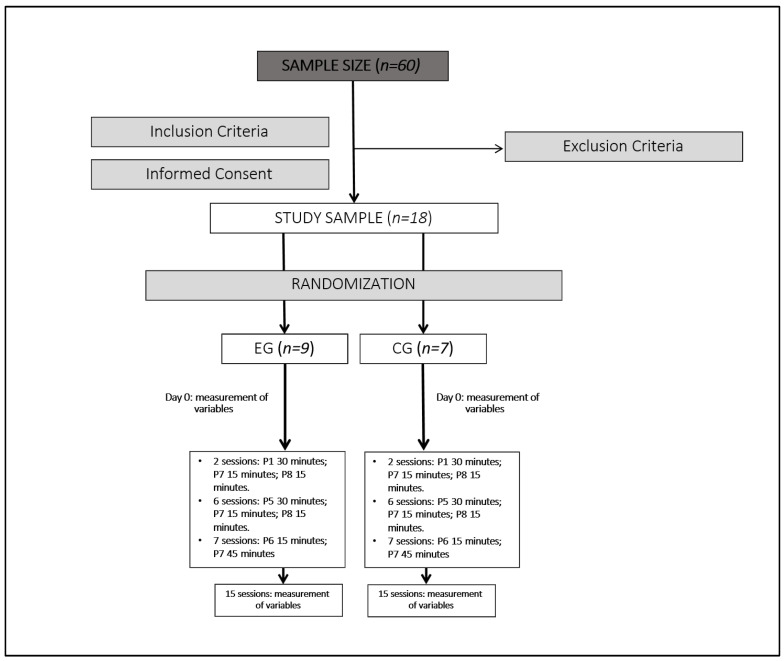
Study intervention algorithm.

Figure 1 shows the study intervention algorithm.

Outcome Measures:

Mechanical Sensitization: Pressure Pain Threshold (PPT) was measured using a Baseline 12-0300.MMT algometer [17] at three sites: C5–C6 vertebral level, D5–D6 dorsal level, and 8 cm distal to the fibular head on the tibialis anterior muscle.

HRV and HR: These were measured using the portable WECARDIO ECG device connected to a smartphone application, validated for COVID-19 patients [18]. Measurements were taken with participants lying down after a five-minute rest period.

Salivary Cortisol: Measured using the Starter Kit IgA/Cortisol LFD with SOMA+ Cube Reader Adapter and Dual Cortisol/Amylase LFD kits (Soma Bioscience Ltd., Wallingford, UK).

Modified Fatigue Impact Scale (MFIS): A self-reported questionnaire assessing physical, cognitive, and psychosocial impacts of fatigue [19].

Pittsburgh Sleep Quality Index (PSQI): A questionnaire evaluating qualitative and quantitative sleep aspects over the past month. It includes 24 questions, 19 of which are self-reported, with the remaining 5 answered by a potential cohabitant. After correction, 7 scores are obtained that report on various aspects of sleep quality: subjective quality, latency (understood as the time the patient believes it takes to fall asleep), duration, habitual efficiency (which assesses the percentage of time the patient believes he is asleep over the total time spent lying down), disturbances (those alterations such as pain, cold, etc.), the use of hypnotics, and daytime dysfunction (presenting as the ease of falling asleep while doing some activity or as greater daytime fatigue) [20].

Health-Related Quality of Life (SF-36): A 35-item questionnaire covering eight domains: physical functioning, physical role, emotional role, social functioning, mental health, general health, bodily pain, and vitality. It also contains an additional element that is not part of any dimension and that measures the declared value of the evolution of health. The Spanish version was used for this study [21].

All questionnaires were self-administered and submitted electronically. These tools are widely used to assess the impact of various conditions on daily life, including post-COVID-19 condition [22].

All variables were measured before intervention (T0), mid-intervention (T1), and post intervention (T2). PPT, HRV, and salivary cortisol were measured at consistent times to account for circadian rhythm effects under controlled temperature and relative humidity. Participants were instructed to remain silent during measurements. For cortisol collection, participants rinsed their mouths with water but avoided brushing their teeth. A collector sponge was placed on the tongue for 50 s until it turned blue. The collector was then placed in a buffer for at least two minutes to mix saliva with the reagent. Two to three drops of the mixture were deposited onto the LFD measurement strip, and results were read after 10 min using the SOMA cube reader. Cortisol measurements were conducted between 6 and 8 a.m., the peak circadian period for cortisol levels [23]. Results were reported in nanomoles per liter (nmol/L) and micrograms per deciliter (μg/dL) for consistency with previous studies.

For statistical analysis, continuous data were summarized as mean and standard deviation. Normality was assessed using the Shapiro–Wilk test. Repeated-measures ANOVA was performed to evaluate the main effects of time on the measured variables, as well as time-by-group and between-group interactions.

Statistical analyses were conducted using SPSS 29.0 (IBM Corp, Armonk, NY, USA, 2023). The significance level was set at *p* < 0.05 with a 95% confidence interval.

## 3. Results

A total of 16 women participated in the study: 9 in the EG and 7 in the CG. No participants were lost during the study. The mean age of the sample was 46.13 (±8.94) years, and the mean weight was 61.81 (±4.06) kg. The results for each group are presented in Table 1.

The results for outcome measures over time, as well as time-by-group and between-group interactions, are shown in Table 2.

Differences in cortisol levels were observed for the time effect and the time-by-group interaction. The EG showed a cortisol concentration 5.04 nmol/L lower than the CG, though this difference was not statistically significant (*p* = 0.699).

The PPT at the C5–C6 level increased significantly over time (*p* = 0.032), with no differences between groups or in time-by-group interactions. For the PPT at the D5–D6 level and tibial site, differences were found in the time-by-group interaction. Specifically, the CG showed a decrease in PPT at T1 and an increase at T2. However, no statistically significant differences were found between groups.

Regarding HRV, both SDNN and rMSSD showed significant differences for the time effect. However, group differences were observed only for SDNN (*p* < 0.05), with no significant time-by-group interaction.

Significant improvements in sleep quality were observed for the time effect, but no differences were found for time-by-group or between-group effects. Fatigue impact improved significantly over time, regardless of group assignment, with no significant time-by-group or between-group differences (Table 2).

Significant changes related with the SF-36 scale were observed over time for the dimensions of bodily pain and vitality, with increases of 13 to 21 points at T2 compared to T0 (*p* < 0.05). No significant differences were found between groups or for time-by-group interactions (Table 3).

Considering the limited sample size, special attention was given to the interpretation of effect sizes (partial eta squared, η^2^) obtained from repeated-measures ANOVA. Moderate to large effect sizes were observed in several variables, such as SDNN (η^2^ = 0.319), MFIS (η^2^ = 0.350), PPT tibial site (η^2^ = 0.323), PSQI (η^2^ = 0.392), and bodily pain (SF-36; η^2^ = 0.321). These values suggest that, despite some non-significant *p*-values due to low statistical power, the magnitude of change may be clinically meaningful and warrants further investigation in larger samples.

## 4. Discussion

The present study aimed to mitigate and improve the variability and inconsistency of symptoms, ranging from mild to severe, associated with manifest DNS. To achieve this, non-invasive neuromodulation was employed as a rehabilitation technique. This therapeutic modality has been previously utilized in studies addressing conditions such as Complex Regional Pain Syndrome (CRPS) [24], neuralgias [25], stroke [26], multiple sclerosis [27], and dementia [28]. However, no prior studies have been identified that employ this technique for post-COVID-19 condition patients.

The present study analyzed variables associated with DNS, such as HRV, salivary cortisol levels, and fatigue, as well as variables related to quality of life, sleep, and pain perception.

Regarding musculoskeletal pain, it has been reported that up to 60% of patients diagnosed with post-COVID-19 condition may experience increased pain sensation in various parts of the body, with the upper and lower extremities being the most prevalent locations [29,30]. The most widely accepted theory to explain this alteration in nociceptive sensation is that systemic immune-inflammatory responses may lead to peripheral and central nervous system excitability through direct and indirect pain pathways [31,32]. The analysis of PPT data revealed that post-COVID-19 condition patients initially exhibited very low sensitivity thresholds before the intervention. Over the course of the sessions, the experimental group showed increased pressure tolerance at the tibial PPT site, while the placebo group exhibited a slight decrease.

Although there is no direct evidence explaining PPT changes in post-COVID-19 condition patients, a study by the Department of Basic and Clinical Sciences at the University of Nicosia explored the relationship between post-COVID-19 condition and fibromyalgia to elucidate shared clinical manifestations such as pain, hyperalgesia, autonomic abnormalities, trigger points, and increased sensitivity [33]. This study by Plaut (2023) delves into fascial and connective tissue damage, which is often overlooked in patients with post-COVID-19 condition [33]. It proposes a neurobiomechanical model to explain the pathogenesis of fibromyalgia and its impact on connective and myofascial tissues. In this context, authors such as Ursini et al. concluded that approximately 30% of the surveyed patients who had recovered from COVID-19 met the diagnostic criteria for fibromyalgia established by the American College of Rheumatology, suggesting that fibromyalgia-like symptoms are common in post-COVID-19 condition [34].

This theory may help explain the low pressure pain threshold (PPT) values observed in patients with post-COVID-19 condition. The biotensegrity model proposed for fibromyalgia suggests that the extracellular matrix’s inability to repair itself, combined with the induction of myofibroblasts, leads to fascial transformation. This ’fascial armoring’ theory [33] aims to uncover the origin of symptoms in this condition, which exhibits immune-rheumatological-psychological-neurological characteristics. The chronic fascial tension described in fibromyalgia may also be present in patients with post-COVID-19 condition.

The findings suggest that COVID-19 causes damage to various tissues, including myofascial tissues, resulting in chronic connective tissue dysfunction and fascial bio-tensegrity impairment, alongside viral persistence and immune-mediated myopathy. While research evaluating PPT in post-COVID-19 condition patients remains sparse, the pre-intervention PPT values observed in both groups in this study align with LC’s unique pathogenesis.

HRV is another critical marker of DNS in post-COVID-19 condition patients. Several studies have demonstrated that HRV values are significantly lower in post-COVID-19 condition patients compared to controls, while mean HR values tend to be higher. At rest, post-COVID-19 condition patients exhibit altered time-domain metrics and reduced non-linear HRV indices, suggesting elevated HR and diminished parasympathetic tone (rMSSD) [35]. The current study’s results partially align with these findings. Over time, both groups exhibited modifications in HRV (SDNN), with a more pronounced decrease in the control group (*p* = 0.021) compared to the experimental group. Mean HR values increased minimally in both groups from baseline (T0), though these changes were not statistically significant over time or between groups. Interestingly, rMSSD values increased from T0 to T2 in the experimental group but decreased in the control group (*p* = 0.193). Notably, a sharp rise in rMSSD was observed in both groups at T1, coinciding with the midpoint of treatment sessions, though the reason for this remains unclear.

Cortisol, a biomarker influenced by DNS, follows a circadian rhythm with peak levels in the morning (6–8 a.m.) and lowest levels at night. This pattern is vital for regulating the sleep–wake cycle, making cortisol measurement a valuable tool for diagnosing sleep disorders and evaluating the impact of therapeutic interventions on circadian rhythms [36]. Salivary cortisol measurement, a stress-free method, has gained popularity [37] for assessing various conditions, including cardiovascular and nervous system disorders, infectious diseases, and psychosocial behavioral changes. Studies have shown that post-COVID-19 condition patients tend to have lower cortisol levels, potentially linked to depressive or anxiety disorders. For instance, Djordje et al. [38] reported approximately 50% lower cortisol levels in post-COVID-19 condition subjects compared to healthy controls. In this study, salivary cortisol levels at baseline fell within the normal range for morning values (0.04–0.56 μg/dL) [39]. Both groups demonstrated significant increases in cortisol levels over time, with a more pronounced improvement in the control group. However, the experimental group’s cortisol concentration increased at T1, whereas the control group’s levels decreased during this period. Cortisol levels exceeding 0.56 μg/dL at 8 a.m. suggest hypercortisolism, while values below 0.04 μg/dL indicate adrenal insufficiency [40].

Regarding self-reported questionnaires on quality of life, fatigue, and sleep quality, the data were inconclusive. The PSQI, which ranges from 0 (“ease of sleeping”) to 21 (“severe difficulty sleeping”), considers scores ≥8 indicative of poor sleep quality [41]. Although sleep problems are prevalent in post-COVID-19 condition patients [42], the initial PSQI scores in this study were not exceedingly high (EG: 12.44; CG 11.14). Both groups showed improvement over 15 treatment sessions (*p* = 0.002), but scores remained above 8, with the control group achieving slightly better scores, though not statistically significant compared to the EG.

In the SF-36 results, both groups showed improvement in bodily pain (*p* = 0.004) and vitality (*p* = 0.026) dimensions, though these changes were not statistically significant. No improvements were observed in other dimensions over time or between groups.

Fatigue is one of the most prevalent symptoms in post-COVID-19 condition patients, affecting 55% [13]. Although no studies have investigated non-invasive neuromodulation for fatigue in post-COVID-19 condition patients, other interventions, such as physical exercise [43] and transcranial electrical stimulation [44], have shown promising and, in some cases, statistically significant results. Fatigue was assessed using the MFIS questionnaire, which ranges from 0 to 84, with a cutoff of 38. MFIS scores decreased by up to 10 points in some cases (EG: T0 60.67 (18.36) to T2 51.78 (22.94); CG: T0 62.71 (9.41) to T2 51.99 (16.39); *p* = 0.002). However, both groups experienced similar reductions, with no significant differences between them (*p* = 0.978).

Although improvements were found across both groups, suggesting non-specific or placebo-mediated effects, the interpretation of partial eta squared values provides further insight. Variables such as SDNN (η^2^ = 0.319), fatigue impact (MFIS; η^2^ = 0.350), and sleep quality (PSQI; η^2^ = 0.392) showed large effect sizes, which may exceed the range typically attributed to spontaneous recovery or placebo responses. Following Cohen and Richardson’s interpretative criteria [45], these effect sizes point toward potentially meaningful changes that may have clinical relevance, even in the absence of significant between-group differences. These findings highlight the importance of including effect size analysis, particularly in small-sample studies, and justify further trials with increased statistical power.

The main limitation of this study is the inability to recruit the estimated sample size. Therefore, further studies with larger cohorts are necessary to definitively assess the efficacy of the non-invasive neuromodulation technique employed. Due to the exploratory nature and small sample size of this pilot study, no formal correction for multiple comparisons (e.g., Bonferroni) was applied. This decision aimed to avoid inflating type II error risk. Instead, we report effect sizes (η^2^) to help interpret the magnitude of changes. Future studies with larger samples will allow for more robust statistical correction procedures.

## 5. Conclusions

While many analyzed variables improved, fulfilling the primary objective of the study, these improvements occurred in both the intervention and placebo groups. Thus, the findings do not confirm that non-invasive neuromodulation, despite being a safe and well-tolerated therapeutic modality for post-COVID-19 condition patients, had a specific positive influence on these changes. Increasing the sample size in future studies is recommended.

## Figures and Tables

**Table 1:** Demographic characteristics of subjects.

	EG	CG	*p*-Value
Age	46.67 (10)	45.43 (8.10)	0.503
Body weight (kg)	61.71 (4.71)	61.94 (3.39)	0.612

EG, experimental group; CG, control group; kg, kilograms.

**Table 2:** Comparison of outcome measures over time and inter groups.

	EG			CG			Time Effects		Time Effects per Group		Inter Groups
	T0	T1	T2	T0	T1	T2	F; *p*-Value	η^2^	F; *p*-Value	η^2^	F; *p*-Value
Cortisol (nmol/L)	6.73 (3.98) 0.24 µg/dL	12.07 (6.89) 0.43 µg/dL	10.10 (5.31) 0.36 µg/dL	7.27 (3.63) 0.26 µg/dL	3.84 (3.29) 0.13 µg/dL	15.14 (11.76) 0.54 µg/dL	4.561; *p* = 0.019	0.246	5.751; *p* = 0.008	0.291	0.155; *p* = 0.699
PPT C5–C6 (kg)	1.43 (0.54)	1.56 (0.68)	1.87 (0.78)	1.52 (0.88)	1.74 (1.74)	1.92 (1.76)	3.902; *p* = 0.032	0.218	0.88; *p* = 0.916	0.006	0.040; *p* = 0.844
PPT D5–D6 (kg)	1.84 (0.84)	2.04 (0.97)	2.27 (0.91)	2.18 (1.71)	2.02 (1.66)	2.37 (2.59)	1.980; *p* = 0.157	0.124	0.555; *p* = 0.580	0.038	0.037; *p* = 0.851
PPT tibial (kg)	2.05 (1.04)	2.33 (1.25)	3.34 (1.26)	2.30 (0.98)	1.67 (1.02)	2.13 (1.56)	6.692; *p* = 0.04	0.323	6.186; *p* = 0.006	0.306	0.344; *p* = 0.064
SDNN (ms)	49.06 (29.31)	149.52 (139.25)	69.68 (58.33)	84.65 (65.81)	185.78 (161.57)	42.26 (23.43)	5.561; *p* = 0.005	0.319	0.579; *p* = 0.567	0.049	0.302; *p* = 0.021
rMSSD (ms)	48.91 (40.64)	188.32 (196.00)	75.15 (80.69)	113.01 (106.69)	243.62 (226.61)	71.00 (68.45)	6.334; *p* = 0.005	0.213	0.389; *p* = 0.682	0.027	0.719; *p* = 0.411
HR (bpm)	80.56 (9.20)	89.56 (20.70)	82.22 (10.69)	71.57 (11.25)	78.86 (37.98)	77.29 (10.93)	0.796; *p* = 0.461	0.054	0.105; *p* = 0.901	0.007	1.871; *p* = 0.193
PSQI	12.44 (5.92)	12.00 (4.69)	9.22 (6.04)	11.14 (2.67)	8.14 (2.48)	8.14 (3.29)	9.044; *p* ≤ 0.001	0.392	2.218; *p* = 0.128	0.137	0.926; *p* = 0.352
MFIS	60.67 (18.36)	55.11 (21.06)	51.78 (22.94)	62.71 (9.41)	54.57 (13.74)	51.99 (16.39)	7.526; *p* = 0.002	0.350	0.168; *p* = 0.846	0.012	0.001; *p* = 0.978

EG, experimental group; CG, control group; T0, before intervention; T1, mid-intervention; T2, post intervention; nmol/L, nanomoles per liter; µg/dL, micrograms per deciliter; PPT, Pressure Pain Threshold; kg, kilograms; SDNN, standard deviation of normal (SDNN) R-R intervals (NN) over a 24 h period; ms, milliseconds; rMSSD, square root of the mean of the differences in the sum of squares between adjacent R-R intervals; HR, heart rate; bpm, beats per minute.

**Table 3:** Comparison of the SF-36 questionnaire based on time and inter groups.

	EG			CG			Time Effects		Time Effects per Group		Inter Groups
SF-36	T0	T1	T2	T0	T1	T2	F; *p*-Value	η^2^	F; *p*-Value	η^22^	F; *p*-Value
Physical function	41.22 (24.05)	45.00 (25.62)	50.49 (28.89)	62.86 (24.64)	65.71 (21.10)	63.75 (20.06)	0.840; *p* = 0.442	0.057	0.0664; *p* = 0.523	0.045	2.608; *p* = 0.129
Physical role	0.00 (0.00)	25.00 (37.50)	25.00 (35.36)	17.85 (37.40)	17.86 (37.40)	9.76 (19.01)	1.445; *p* = 0.253	0.094	2.642; *p* = 0.089	0.159	0.014; 0.909
Body pain	22.44 (11.76)	38.44 (23.56)	36.00 (21.82)	28.57 (20.59)	43.14 (23.92)	50.48 (20.67)	6.630; *p* = 0.004	0.321	0.501; *p* = 0.611	0.035	1.001; *p* = 0.334
General health	26.11 (12.18)	23.89 (11.67)	28.78 (12.23)	34.29 (12.67)	33.57 (12.20)	33.19 (14.23)	0.753; *p* = 0.480	0.051	1.053; *p* = 0.362	0.070	1.335; *p* = 0.267
Vitality	25.00 (19.84)	29.44 (24.55)	31.67 (10.16)	15.00 (14.43)	32.85 (14.10)	24.81 (14.65)	4.156; *p* = 0.026	0.229	1.528; *p* = 0.234	0.098	0.291; *p* = 0.598
Social function	34.72 (32.24)	44.44 (33.14)	47.22 (39.41)	42.86 (26.86)	42.86 (35.25)	51.67 (39.81)	1.039; *p* = 0.367	0.069	0.220; *p* = 0.804	0.015	0.057; *p* = 0.814
Emotional role	63.00 (48.43)	51.86 (44.49)	48.15 (47.46)	62.00 (44.85)	57.16 (41.78)	60.00 (40.55)	0.386; *p* = 0.684	0.027	0.177; *p* = 0.839	0.012	0.081; *p* = 0.780
Mental health	51.11 (19.98)	50.67 (29.25)	52.88 (24.25)	59.43 (10.18)	64.57 (17.26)	63.35 (19.44)	0.300; *p* = 0.743	0.021	0.257; *p* = 0.775	0.018	1.222; *p* = 0.288

EG, experimental group; CG, control group; T0, before intervention; T1, mid-intervention; T2, post intervention.

## Data Availability

Data available under demand. The data are not publicly available due to privacy concerns regarding the study participants.
