# Correction: Melián-Ortíz et al. Superficial Neuromodulation in Dysautonomia in Women with Post-COVID-19 Condition: A Pilot Study. *Brain Sci.* 2025, *15*, 510

**DOI:** 10.3390/brainsci15070719

**Published:** 2025-07-04

**Authors:** Alberto Melián-Ortíz, Eduardo Zurdo-Sayalero, Sara Perpiñá-Martínez, Antonio Delgado-Lacosta, Carmen Jiménez-Antona, Josué Fernández-Carnero, Sofía Laguarta-Val

**Affiliations:** 1 Faculty of Nuring and Physiotherapy Salus Infirmorum, Universidad Pontificia de Salamanca, 28015 Madrid, Spain; amelianor@upsa.es (A.M.-O.); ezurdosa@upsa.es (E.Z.-S.); sperpinama@upsa.es (S.P.-M.); ardelgadola@upsa.es (A.D.-L.); 2 Department of Physical Therapy, Occupational Therapy, Rehabilitation and Physical Medicine, Faculty of Health Sciences, Universidad Rey Juan Carlos (URJC), 28922 Alcorcón, Spain; carmen.jimenez@urjc.es (C.J.-A.); sofia.laguarta@urjc.es (S.L.-V.); 3 Cognitive Neuroscience, Pain and Rehabilitation Research Group (NECODOR), Faculty of Health Sciences, Universidad Rey Juan Carlos (URJC), 28933 Madrid, Spain

## Affiliation Change

In the original publication, the affiliation was NEDECOR, which will be replaced by NECODOR. The reason was a typographical error. It should read NECODOR because the acronym CO stands for Cognitive, as it corresponds to the expanded name of the research group of some of the authors of the paper.

## Text Correction

In the original publication [1], a correction was made to the fourth paragraph of the Discussion section, which stated “Approximately 30% of post-COVID-19 condition patients share clinical characteristics with fibromyalgia [34]. This study by Clauw et al. delves into the often-overlooked fascial and connective tissue damage in post-COVID-19 condition patients, proposing a neurobiomechanical model to explain fibromyalgia’s pathogenesis and its impact on connective and myofascial tissues. This theory could help explain the low PPT values observed in post-COVID-19 condition patients. The biotensegrity model proposed for fibromyalgia suggests that the extracellular matrix’s inability to repair itself, coupled with the induction of myofibroblasts, leads to fascial transformation. This “fascial shielding” theory aims to uncover the origins of symptoms in this immune-rheumatological-psychological-neurological disease. The chronic fascial tension described in fibromyalgia might also be present in post-COVID-19 condition patients.”

The revised paragraph reads as follows:

“This study by Plaut (2023) delves into fascial and connective tissue damage, which is often overlooked in patients with post-COVID-19 condition [33]. It proposes a neurobiomechanical model to explain the pathogenesis of fibromyalgia and its impact on connective and myofascial tissues. In this context, authors such as Ursini et al. concluded that approximately 30% of the surveyed patients who had recovered from COVID-19 met the diagnostic criteria for fibromyalgia established by the American College of Rheumatology, suggesting that fibromyalgia-like symptoms are common in post-COVID-19 condition [34].

This theory may help explain the low pressure pain threshold (PPT) values observed in patients with post-COVID-19 condition. The biotensegrity model proposed for fibromyalgia suggests that the extracellular matrix’s inability to repair itself, combined with the induction of myofibroblasts, leads to fascial transformation. This ’fascial armoring’ theory [33] aims to uncover the origin of symptoms in this condition, which exhibits immune-rheumatological-psychological-neurological characteristics. The chronic fascial tension described in fibromyalgia may also be present in patients with post-COVID-19 condition.”

## Reference

The original reference [34] “Clauw, D.J.; Calabrese, L. Rheumatology and Long COVID: Lessons from the study of fibromyalgia. *Ann. Rheum. Dis.* **2024**, *83*, 136–138. https://doi.org/10.1136/ard-2023-224250” has been replaced with the following reference:

34. Ursini, F.; Ciaffi, J.; Mancarella, L.; Lisi, L.; Brusi, V.; Cavallari, C.; D’Onghia, M.; Mari, A.; Borlandelli, E.; Faranda Cordella, J.; et al. Fibromyalgia: A new facet of the post-COVID-19 syndrome spectrum? Results from a web-based survey. *RMD Open* 2021, *7*, e001735. https://doi.org/10.1136/rmdopen-2021-001735.

This correction does not affect the numerical order of the remaining references in the bibliography.

The authors state that the scientific conclusions are unaffected. This correction was approved by the Academic Editor. The original publication has also been updated.
